# Effect of Acute Exercise on Prostate Cancer Cell Growth

**DOI:** 10.1371/journal.pone.0067579

**Published:** 2013-07-05

**Authors:** Helene Rundqvist, Martin Augsten, Anna Strömberg, Eric Rullman, Sara Mijwel, Pedram Kharaziha, Theocharis Panaretakis, Thomas Gustafsson, Arne Östman

**Affiliations:** 1 Department of Oncology and Pathology, Karolinska Institutet, Stockholm, Sweden; 2 Department of Medical Laboratory Sciences and Technology, Karolinska Institutet, Stockholm, Sweden; Clermont Université, France

## Abstract

Physical activity is associated with reduced risk of several cancers, including aggressive prostate cancer. The mechanisms mediating the effects are not yet understood; among the candidates are modifications of endogenous hormone levels. Long-term exercise is known to reduce serum levels of growth stimulating hormones. In contrast, the endocrine effects of *acute* endurance exercise include *increased* levels of mitogenic factors such as GH and IGF-1. It can be speculated that the elevation of serum growth factors may be detrimental to prostate cancer progression into malignancy. The incentive of the current study is to evaluate the effect of acute exercise serum on prostate cancer cell growth. We designed an exercise intervention where 10 male individuals performed 60 minutes of bicycle exercise at increasing intensity. Serum samples were obtained before (rest serum) and after completed exercise (exercise serum). The established prostate cancer cell line LNCaP was exposed to exercise or rest serum. Exercise serum from 9 out of 10 individuals had a growth inhibitory effect on LNCaP cells. Incubation with pooled exercise serum resulted in a 31% inhibition of LNCaP growth and pre-incubation before subcutaneous injection into SCID mice caused a delay in tumor formation. Serum analyses indicated two possible candidates for the effect; increased levels of IGFBP-1 and reduced levels of EGF. In conclusion, despite the fear of possible detrimental effects of acute exercise serum on tumor cell growth, we show that even the short-term effects seem to add to the overall beneficial influence of exercise on neoplasia.

## Introduction

Prostate cancer is the second most frequent cancer diagnose in men in the world today. The highest incidence rates are found in the developed western countries and are 20 fold higher than the incidence rates found e.g. in South Central Asia and western Africa [1]. The discrepancy is partly due to the established use of PSA testing but lately a significant impact of life-style effects are being recognized [2]. Physical activity is an adjustable life-style factor associated with a reduced risk of several cancers, including prostate cancer [3].

A recent meta analysis comprising studies until 2012 suggests that being physically active is associated with a modest but significant reduction in risk of prostate cancer [4]. In addition, studies examining physical activity in relation to high-grade prostate cancer and prostate cancer mortality also reported a significant risk reduction [5]–[7].

The mechanisms mediating the effects of physical activity are not yet understood, although some candidates including weight control, improved immune cell function and modifications of endogenous hormone levels such as leptin, insulin and insulin like growth factor -1 (IGF-1) have been put forward [8]. Elevated serum levels of leptin, insulin and IGF-1 are all associated with high risk of prostate cancer incidence and progression [8]–[12] and long-term exercise is known to reduce serum levels of these and other endogenous hormones [13], [14].

Serum from endurance trained individuals on a low-fat, high-fiber diet has been shown to inhibit growth of an established prostate cancer cell line when compared to control serum [15]. More recent studies from the same group suggest that the mechanism behind the effect is mediated through the IGF-1 axis [16].

In contrast to long-term exercise, the endocrine effects of acute endurance exercise include *increased* levels of mitogenic factors such as growth hormone (GH) [17], various cytokines [18] including IL-6 [19], [20] and also increased bioavailability of IGF-1 [21], [22].

It can be speculated that the increase in serum growth factors induced by acute exercise may be detrimental to prostate cancer progression into malignancy. The incentive of the current study is to evaluate the effect of acute exercise serum on prostate cancer cell growth.

## Methods

### Ethics Statement

Prior to the human exercise study, the experimental protocol was explained to all subjects and written, informed consent was obtained. The study was approved by the Ethics Committee of Karolinska Institutet (266/01) and conformed to the *Declaration of Helsinki*. The animal experiments were conducted in accordance with national guidelines and approved by the Stockholm North Ethical Committee on Animal Experiments (N19/08), all efforts were made to ameliorate suffering of the tumor bearing animals.

### Experimental Model for the Acute Exercise Study

Ten healthy male subjects were included in the study. Their median (range) age, height and weight were 25 (18–37) yrs, 180 (170–190) cm and 77 (58–82) kg, respectively. Median (range) maximal oxygen uptake (VO2-max) determined prior to the experiments was 3.7 (3.1–4.3) L*min^−1^. The exercise was performed on an electro-dynamically loaded cycle ergo meter. The subjects performed about 65 min of exercise. For the first 20 min, subjects cycled at 60 rpm at a median (range) work rate of 125 (105–148) watts (W), chosen to correspond to 50% of VO2-max, after which the work rate was increased to 165 (138–195) W, corresponding to 65% of VO2-max for another 40 min. Teflon catheters were inserted into both femoral veins and the femoral artery, at the level of the inguinal ligament. Blood samples were drawn simultaneously from the femoral artery (fa) and the ipsilateral femoral vein (fv) as described in [23]. In short, resting samples and samples collected 120 min after the end of exercise were used for further analyses.

### Growth of Prostate Cancer Cells

Growth rates of the cell lines NIH 3T3 and LNCaP cells, previously used in Augsten et al [24]; a total of either 2×10^3^ fibroblasts or 5×10^4^ LNCaP cells were seeded in 96-well plates. Fibroblasts were grown in DMEM supplemented with 5% FCS and either 5% rest or exercise serum, whereas LNCaP cells were cultured in RPMI 1640 medium supplemented with 5% FCS and either 5% rest or exercise serum for 24, 48 and 96 hours. 2×10^4^ 22rv1 and Du145 cells, previously used in [25] were cultured in RPMI 1640 medium supplemented with 5% FCS and either 5% rest or exercise serum for 96 hours. Cell numbers were determined by fixating the cells with 4% PFA for 20 minutes, followed by incubation with crystal violet (Chroma, Stuttgart, FRG) solution (0.1%, w/v, with ethanol 2%, v/v in 0.5 M Tris-C1, pH 7.8; 100 µl per well) for 20 min at room temperature. The stained cell layer was rinsed thoroughly with tap water, air dried and incubated with SDS solution (0.1%, w/v, with ethanol 50%, v/v, in 0.5 M Tris-C1, pH 7.8; 100 µL per well) for 30 min at room temperature. Meanwhile crystal violet was completely released from the cells into the supernatant. The supernatant was scanned in a DU Series 70 Beckman spectrophotometer (Beckman Coulter, Fullerton, CA, USA) and read at a fixed wavelength of 600 nm.

### Apoptosis and Proliferation Assays

Redistribution of plasma membrane phosphatidylserine is a marker of apoptosis and was assessed with annexin-V-fluos staining kit (Roche Molecular Biochemicals, Mannheim. Germany). Briefly, 2×10^5^ LNCaP cells were incubated with exercise or rest serum for 24 and 48 hours, collected, washed in PBS, pelleted and re-suspended in incubation buffer (10 mM HEPES/NaOH, pH 7.4, 140 mM NaCl, 5 mM CaCl_2_) containing 1% Annexin V and PI. Samples were incubated for 10 min before analysis on a fluorescence-activated cell sorter (FACS) Calibur flow cytometer (Becton Dickinson, Franklin Lakes, NJ, USA) using Cell Quest software (San Jose, CA, USA). For proliferation assessment, Click-iT EdU Microplate Assay kits were used (Invitrogen. Eugene, OR, USA). In short, LNCaP cells were seeded in a 96 well plate at 5×10^3^ cells per well and allowed to settle overnight. EdU stock solution was prepared and diluted in exercise or rest media at a working concentration of 10 µM, 100 µL of supplemented media was added to the cells and allowed to incubate for 24 hours. Cell fixation and labeling was done according to manufacturers protocol. Quantitation and analysis was done on a VICTOR2 Multilabel Counter (PerkinElmer, Waltham, MA, USA).

### Xenograft Model of Tumor Growth

Xenograft tumors were generated by incubation of LNCaP cells in medium supplemented with rest serum or exercise serum for 48 hours and NIH-3T3 cells in medium supplemented with rest serum. Cells were trypsinized, re-suspended in medium, and counted with a cell counter (Chemometec, Allerod, Denmark). For co-injection analysis, 2×10^6^ LNCaP cells and 0.5×10^6^ NIH-3T3 cells were washed, combined, and re-suspended in 100 µL of cold PBS. Subsequently, the 100 µL cell suspensions were injected subcutaneously in the right lateral flank of 7–8 week-old, male SCID mice (strain CB17sc) from Taconic, DK, n = 10 per group. Animals were monitored daily, and tumor size measured every 3–4 days using external caliper. Experiments were terminated when individual tumors reached a volume of 1 cm^3^ or at day 49. Tumors were excised and split before either fixation in 4% paraformaldehyde overnight at 4°C followed by transfer to 70% ethanol, or snap frozen in liquid nitrogen.

### Analysis of Proliferation and Apoptosis in Tumor Samples

Tissue sections from xenograft endpoint tumors were de-paraffinized and rehydrated in xylene (3×5 min), 99% ethanol (2×2 min), 96% ethanol (2×5 min), 70% ethanol (1×2 min), and then washed in distilled H2O. For apoptosis, the DeadEnd™ Colorimetric TUNEL System was used according to manufacturers instructions (Promega Corporation, Madison, WI, USA). For assessment of proliferation a PCNA histochemistry approach was used; antigen retrieval was done in 2 full racks of slides, boiled in a microwave oven 2×7 min at 650W in 10 mM citric acid buffer, pH 6.0. Sections were then left to cool for at least 30 min before being washed in PBS with 0.1% Tween-20 (PBT). Endogenous peroxidase activity was quenched by incubation in PBT containing 3% H_2_O_2_ for 10 min, then washed in PBT (3×5 min). Slides were blocked in 20% goat serum diluted in PBT for 30 min and incubated overnight at 4°C with PCNA antibody (M0879 clone PC10, Dako Cytomation, (Dako Nordic a/s, Glostrup, Denmark) diluted 1∶100 in 20% goat serum diluted in PBT. After washing in PBT, slides were incubated with biotinylated goat anti-mouse antibody (E0432, 1∶500, Dako Cytomation) for 45 min at RT following incubation with the ABC kit (SK6100, Vectastain ABC-HRP, Vector Laboratories, Burlingame, CA, USA) for 45 min after washing in PBT. The signal was developed using diaminobenzidine hydrochloride, DAB (SK4100, Vector Laboratories), and sections were counter- stained with Mayer’s Heamatoxylin (Histolab Products AB, Gothenburg, Sweden), followed by washing in H_2_O, dehydration in ethanol (70%–95%–99%) and xylene. The slides were then mounted with Mountex mounting medium. Pictures from 5 different, randomly selected vision-fields per tumor were taken and the number of PCNA or tunnel positive structures was analyzed with ImageJ software.

### Serum Analysis

Pools of exercise serum from 10 individuals and corresponding rest serum were analyzed with a human protein growth factor array kit (RayBiotech, Norcross, GA, USA). 200 µL of pooled exercise and rest serum was diluted with 1× blocking serum (provided in the kit) while the membranes were blocked for 1 hour in the same blocking buffer. 1 ml of diluted serum was added and incubated at room temperature for 2 hours. The membranes were then analyzed according to the manufacturer's instructions. Chemiluminiscens detection was done on the ImageQuant LAS 4000 (GE Healthcare, Buckinghamshire, UK). Density of individual spots was quantified with Image J software. Individual serum concentrations of EGF and IGFBP1 were determined using a sandwich enzyme-linked immunoassay (ELISA; Abcam, Cambridge, UK) according to the manufacturer’s directions. The minimum detectable levels of these kits were 0.82 pg/mL for EGF and 2.74 pg/mL for IGFBP1.

### Statistics

Student’s t-test was used to compare the effects of pre-exercise serum with effects of post exercise serum. A two way ANOVA was used to compare tumor growth between the two groups. Planned comparison was used (i.e., post hoc test) to identify significant interactions in the ANOVA model. Differences were considered significant at p<0.05. Unless otherwise stated, data is presented as mean ± SEM.

## Results

### Exercise Serum Reduce Growth of a Prostate Cancer Cell Line *in vitro*

To investigate the effect of acute exercise on prostate cancer cell growth we designed an exercise intervention where 10 male individuals performed 60 minutes of two-legged bicycle exercise at increasing intensity. Through femoral catheterization both arterial and venous blood were obtained and serum was extracted. Since we address the question of systemic effects, arterial serum taken before (rest serum) and after exercise (exercise serum) was used for the analyses. The established cell line LNCaP was chosen as a prostate cancer model based on its naïve p53 activity and moderate tumor formation capacity, thereby allowing for a promoting or inhibitory effect to protrude. The tumor supporting capacity of LNCaP cells can be enhanced by co-injection of stromal cells, e.g. NIH3T3 fibroblasts [26]. Both LNCaP and NIH3T3 cells were incubated for 48 hours *in vitro,* in media supplemented with rest or exercise serum from the 10 individuals separately.

**Figure 1:**
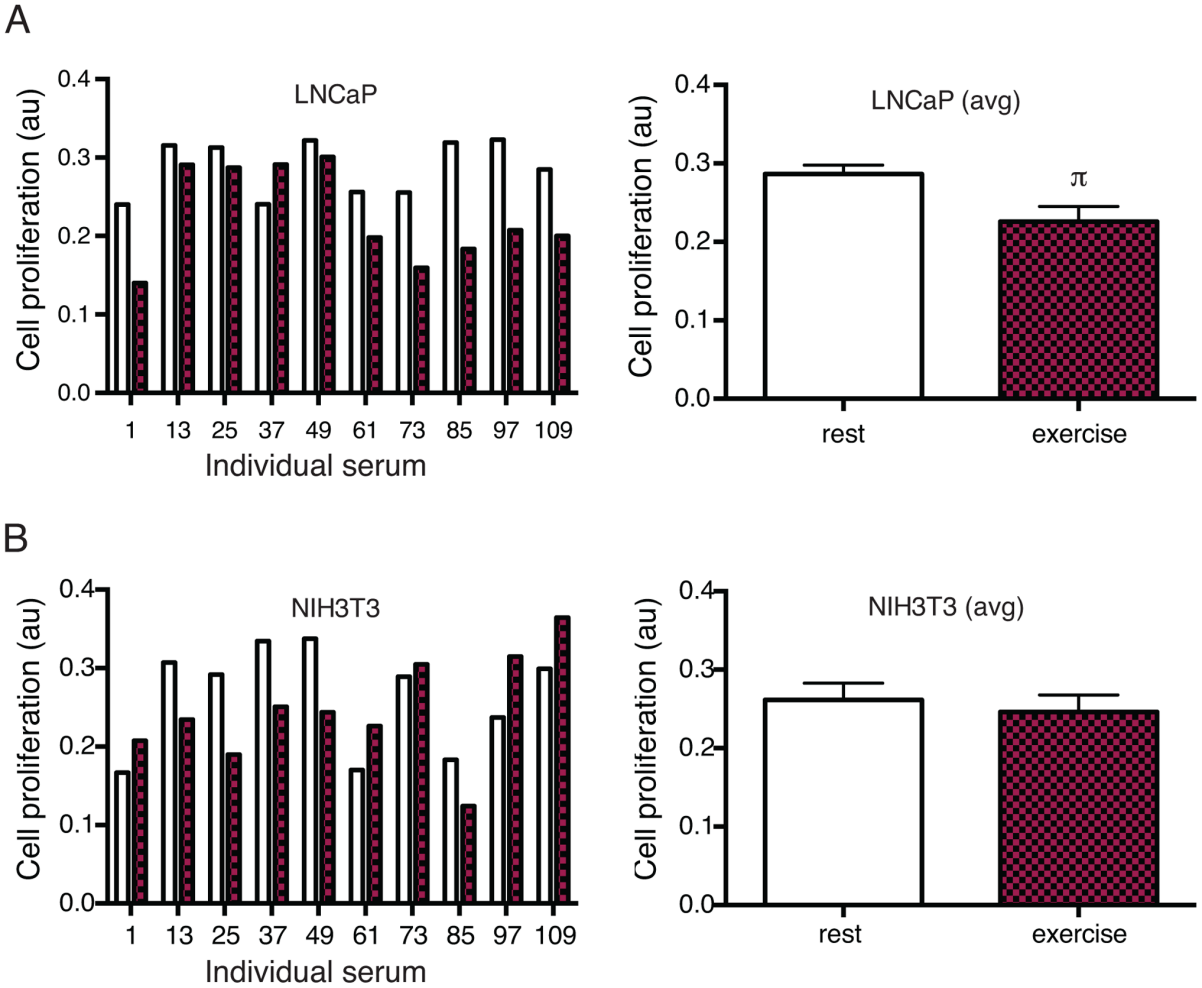
Growth of prostate cancer cells is reduced when exposed to exercise serum from 9 out of 10 individuals. A) Effect on LNCaP cells incubated for 48 hours with resting (rest) and exercise serum (exercise) from 10 individuals separately. B) Effect of the 10 individual serums on NIH3T3 cells. Data show all individuals separately (left panel) and as mean ± SEM. au (arbitrary units). π denotes a significant (p≤0.05) difference between incubation with rest and exercise serum.

**Figure 2:**
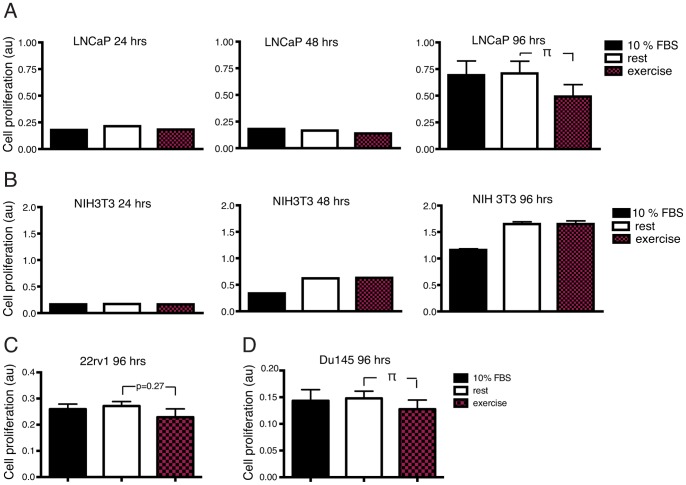
Incubation with a pool of exercise serum reduce growth of prostate cancer cells *in vitro.* A) Effect of 24, 48 and 96 hours incubation with normal media supplemented with 10% FCS (normal), media supplemented with a pool of human serum from 10 resting individuals (rest) or serum from the same individuals after a single bout of exercise (exercise) on growth of the prostate cancer cell line LNCaP. B) Effect of incubation under the same conditions as in A) on growth of the mouse fibroblast cell line NIH3T3. C) Effect of 96 hours incubation with respective serum on the prostate cancer cell lines 22rv1 and Du145. Data is presented as mean ± SEM. au (arbitrary units). π denotes a significant (p≤0.05) difference between incubation with rest and exercise serum.

Exercise serum from 9 out of 10 individuals had a growth inhibitory effect on LNCaP cells after 48 hours incubation (figure 1A and B) compared to incubation with the corresponding rest serum. Growth of NIH3T3 cells was increased by 5 individual exercise serums and reduced by 5 (figure 1C and D). Incubation of LNCaP cells with pooled exercise serum from 10 individuals for 96 hours resulted in a 31% inhibition of tumor cell growth (p<0.05) (Figure 2A, top panel) compared to incubation with a pool of rest serum. The effect on prostate cancer cells was validated in two additional low malignant prostate cancer cell lines, Du145 and 22rv1. Growth of Du 145 was significantly reduced after 96 hour exposure to exercise serum, 22rv1 showed a trend towards reduced growth. NIH3T3 cells grew equally well in pools of exercise and rest serum (figure 2A, bottom panel).

Thus, data show that incubation with exercise serum did not increase growth of prostate cancer cells *in vitro*, but rather had a consistent growth inhibiting effect when compared to serum from the same individual at rest. The effect was found in analyses of the individual serums as well as when comparing a pool of exercise serum to a pool of rest serum. Exercise serum did not show any growth promoting or inhibitory effect on NIH3T3 fibroblasts, suggesting that the effect of exercise serum is specific for cancer cells and not growth inhibitory on cultured cells in general (figure 1B and 2B).

### Exercise Serum Reduces Tumor Cell Growth by Inhibiting Proliferation

To analyze if the growth inhibitory effect of exercise serum on LNCaP prostate cancer cells was due to increased apoptosis and/or reduced proliferation, AnnexinV/PI flow FACS (figure S1A) and an EdU incorporation assay was used.

**Figure 3:**
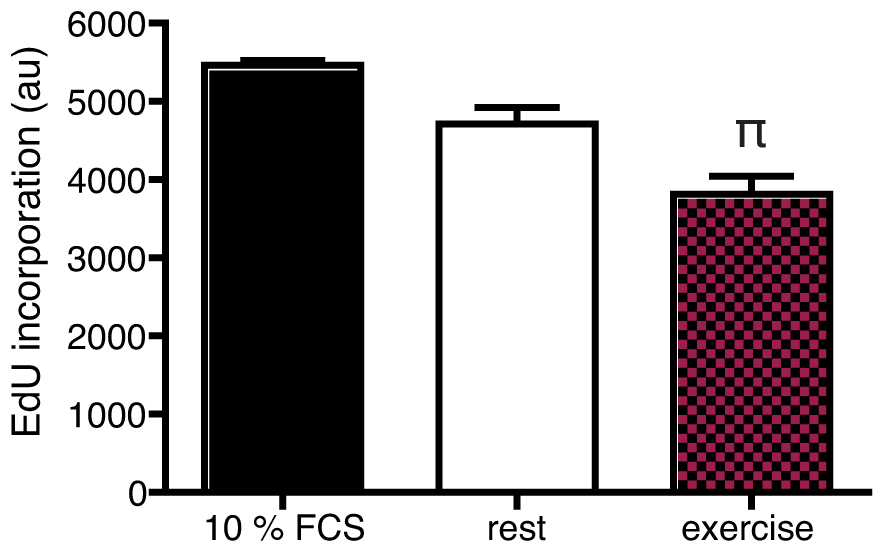
Incubation with exercise serum reduce tumor cell growth by inhibition of proliferation. EdU incorporation in LNCaP cells exposed to normal, rest and exercise serum for 24 hours. Data is presented as mean ± SEM of 4 consecutive experiments. au (arbitrary units). π denotes a significant (p≤0.05) difference between incubation with rest and exercise serum.

Assessment of apoptosis after 24 and 48 hours incubations showed that the fraction of apoptotic cells did not differ between LNCaP cells incubated with exercise serum and cells incubated with rest serum (figure S1B and S1C). However, analyses of proliferation showed that EdU incorporation was significantly reduced in cells incubated with exercise serum for 24 hours (figure 3).

These data indicate that the reduced growth in response to acute exercise serum is due to inhibition of proliferation rather than stimulation of apoptosis and that the effect is present already after 24 hours of incubation although significantly manifested in the growth assay after 96 hours.

### Pre-incubation with Exercise Serum Delays *in vivo* Tumor Formation

We wanted to test if pre-incubation with exercise serum would affect tumor growth of LNCaP cells *in vivo*. LNCaP cells alone have limited tumor-forming capacity when injected subcutaneously. However their tumor supporting capacity can be markedly enhanced by co-injection of stromal cells such as NIH3T3 fibroblasts [26].

**Figure 4:**
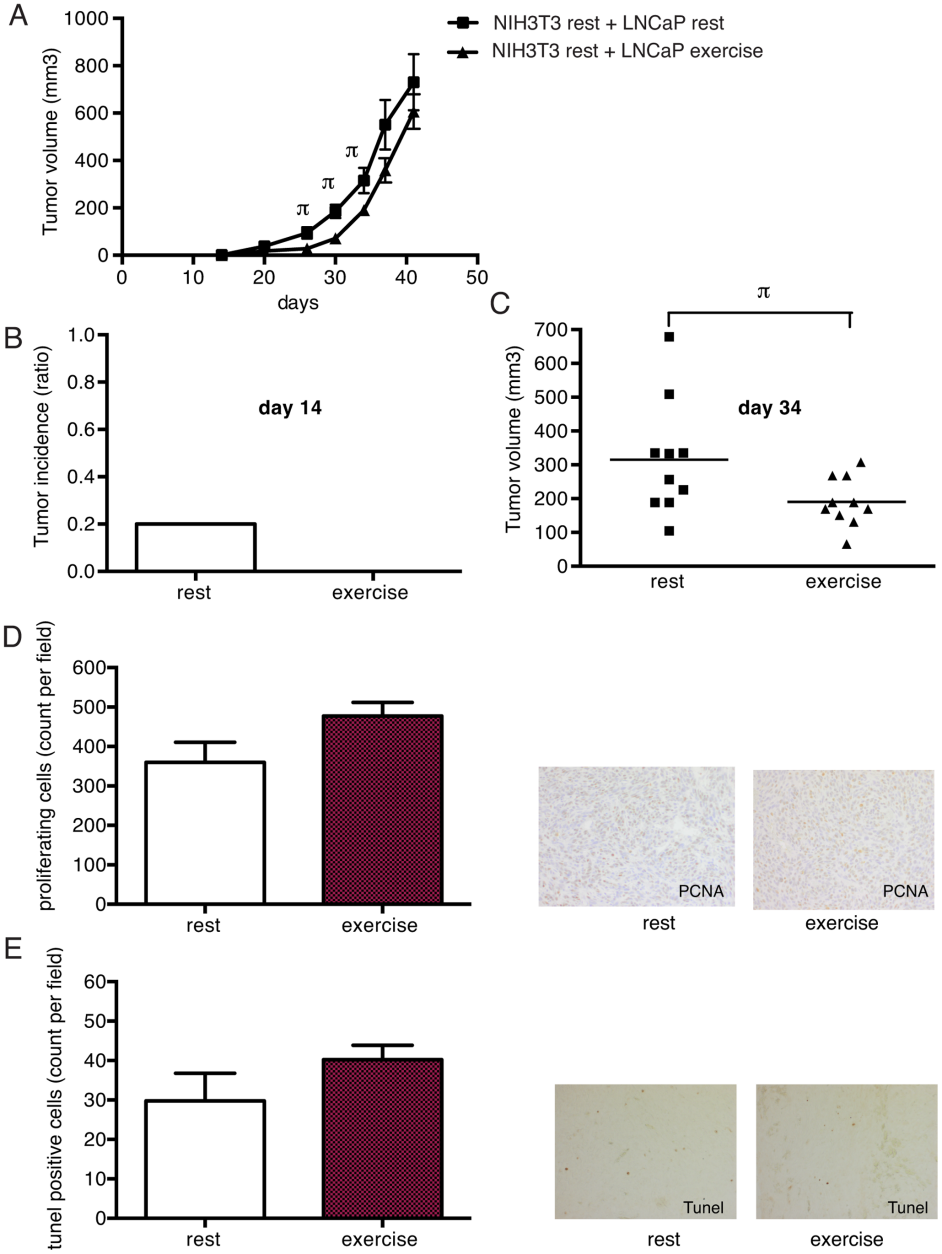
Pre-injection of tumor cells with exercise serum delays onset of tumor growth. 2×10^6^ LNCaP cells incubated for 48 hours in either rest serum or exercise serum were co-injected with NIH3T3 cells (4∶1) subcutaneously in SCID mice. A) Tumor growth curves of cells preincubated with rest and exercise serum respectively. Significant (p≤0.05) differences are denoted with π. n = 10 animals per group. B) Tumor incidence (percent of mice carrying tumors) at day 14 in the rest and exercise group. C) Scatter plot of tumor volume in rest and exercise group at day 34 after injections. D) Proliferating cells in tumors after experimental endpoint assessed by expression of proliferating cell nuclear antigen (PCNA). E) Apoptotic cells in tumors after experimental endpoint, assessed by Tunel stainings. For D) and E) data is the nr of positive cells per field, shown as mean ± SEM.

LNCaP and NIH3T3 cells were incubated for 48 hours *in vitro* in media supplemented with rest or exercise serum from the 10 individuals and then co-injected (4∶1) subcutaneously in SCID mice. Mice injected with exercise serum incubated LNCaP cells showed no tumor incidence at day 14 after injection, while control tumors from rest serum showed 20% incidence (figure 4A and B). The delay in tumor formation persisted throughout the experiment and tumor growth curves were significantly different between the two groups (figure 4A and C). However, once the tumors were established there seemed to be no difference in growth rate.

To confirm the notion that the tumors grew equally well once they were established we analyzed tumors from both groups for proliferation and apoptosis.

Whole tumor samples were harvested and fixated at the endpoint of the *in vivo* experiment and analyzed for apoptosis by looking at DNA fragmentation. Proliferation was assessed by immunohistochemical staining for proliferating cell nuclear antigen (PCNA) expression.

There was no significant difference between the groups at the end of the *in vivo* experiment with regard to either proliferation (figure 4D) or apoptosis (figure 4E).

The data supports the notion that the initial *in vivo* effect of pre-incubation with exercise serum, is lost over time. This is most likely because the exercise stimulus is absent once the cells are injected into mice. However, taken together the data suggests that the effect of exercise serum on tumor cells *in vitro* can be translated to the *in vivo* situation.

### EGF and IGFBP-1 are Candidates for Exerting the Inhibitory Effect of Exercise Serum

**Figure 5:**
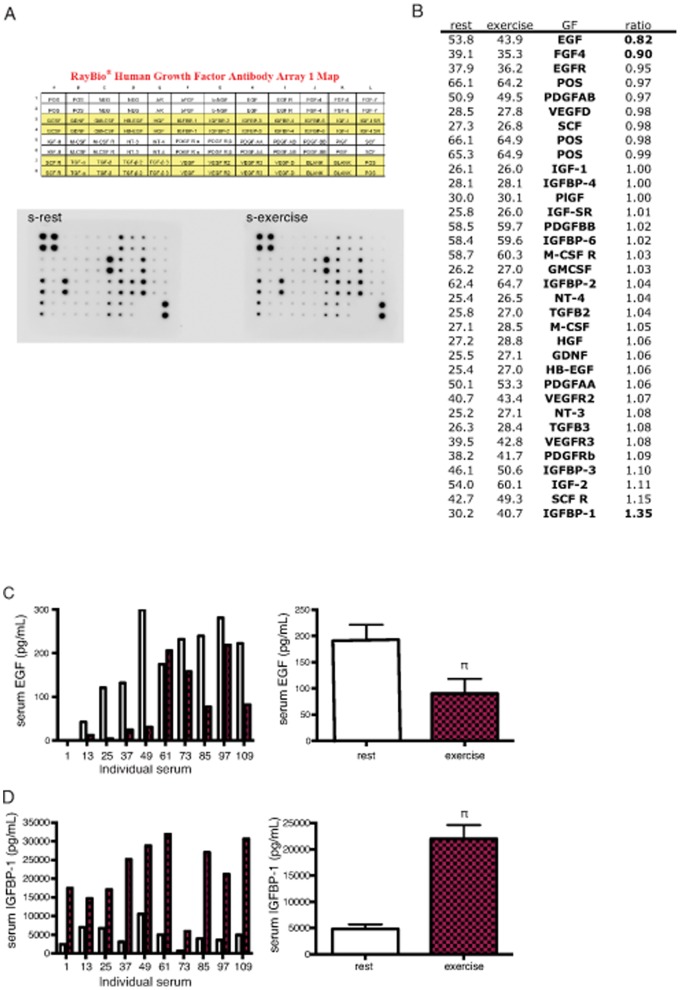
EGF and IGFBP-1 are candidates for exerting the inhibitory effect of exercise serum on tumor cell growth. A) Analysis of growth factor content in rest and exercise serum using a growth factor array approach. B) Rest/exercise serum ratio of 42 growth factors and cytokines, analyzed in multiplex. C) ELISA analysis of individual (left panel) and average (right panel) levels of serum EGF in rest and exercise serum, n = 9. D) ELISA analysis of individual (left panel) and average (right panel) levels of serum IGFBP-1 in rest and exercise serum, n = 10. Data is presented as individual levels or mean ± SEM. π denotes a significant (p≤0.05) difference between incubation with rest and exercise serum.

In order to identify candidate factors responsible for the growth inhibition seen with exercise serum we used a growth factor protein array approach. Forty-two growth factors and cytokines were analyzed in multiplex and shown as exercise serum/rest serum ratios (figure 5A). In addition, serum levels of cortisol were analyzed.

Data brought out two independent candidates. Epidermal growth factor (EGF), was the factor showing the lowest levels in exercise serum when compared to rest serum (figure 5B). The factor with the highest levels in exercise serum compared to rest serum was Insulin like growth factor binding protein 1 (IGFBP-1) (figure 5B). IGFBP-1 showed an increase of 35% compared to serum taken before exercise, EGF show 18% lower levels after exercise. To validate these findings, ELISA was run on individual samples. In the ELISA analysis IGFBP-1 levels were up by approximately 4.6 times, in a highly significant fashion (figure 5D) while EGF levels were reduced by 43% (figure 5C). In addition, cortisol levels increased during exercise (60 minutes time point) but had returned to pre-exercise levels when the exercise serum was harvested (figure S2A).

Both EGF and IGF-1 are linked to prostate cancer risk. Bioavailability of IGF-1 is dependent on the abundance of its binding proteins, IGFBP1–6.

## Discussion

Prostate cancer develops over a long period of time with early PIN lesions that may or may not progress into prostate cancer. It is believed that this transition from benign hyperplasia to malignant carcinoma is affected by lifestyle factors. Previous studies suggest that serum from individuals that have been active over long periods of time may exert an inhibitory effect on prostate tumor cell growth *in vitro* [27]. The present study is the first to evaluate the *acute* serum effects of endurance exercise on prostate cancer cell growth. In the current study we present novel data showing that serum obtained directly after a bout of strenuous exercise, despite its mitogenic potential, reduced proliferation of prostate cancer tumor cells. The effect on prostate cancer cell growth was significant both in comparisons of pools of serum from 10 individuals and in comparisons of the individual serum pairs.

Acute exercise serum had no apoptotic effect on the tumor cells. This is in contrast with the effect of chronic exercise serum described by Barnard et al. where apoptosis was induced through p53 activation [16]. Rather than inducing apoptosis we found that acute exercise serum altered the rate of active DNA synthesis measured by incorporation of EdU, indicating that the inhibitory effect is primarily on tumor cell proliferation.

Interestingly, the effect on tumor cells *in vitro* is robust enough to be extrapolated to the *in vivo* situation. Exercise serum-treated LNCaP cells initially displayed impaired tumor growth compared to LNCaP cells pre-incubated in rest serum. As would be expected, the inhibitory effect seemed to be reversible and was lost once the xenografts were established. In line with this, we found no difference in the number of proliferating or apoptotic cells when tumors were harvested.

From the growth factor array, two factors correlated to the growth data and emerged as possible candidates to convey the exercise effect; reduced levels of EGF and increased levels of IGFBP-1. EGF is known to stimulate the growth of LNCaP cells [28]. Increased EGF receptor expression is found in prostate cancer and is associated with poor prognosis [29]. Compounds inhibiting EGF receptor activity in prostate cancer are currently undergoing clinical trials [30]. EGF is traditionally considered to be released in a paracrine fashion from the local microenvironment and not provided through circulation. Whether circulating levels of EGF is of clinical relevance to prostate cancer progression has, to our knowledge, not been investigated.

The IGF binding proteins modulates the bioavailability of IGF-1 and 2. IGF-1 is known to regulate e.g. cell proliferation and apoptosis [31] and high levels of circulating IGF-1 have been associated with increased prostate cancer risk [11], [32]. It has previously been shown that serum from long-term endurance trained individuals exerts an inhibitory effect on LNCaP growth, attributed to low levels of IGF-1 and high levels of IGFBP-1. [16], [33], [34]. In the studies mentioned, addition of IGFBP-1 to the control serum reduced tumor cell growth to the same extent as exercise serum.

In studies of acute exercise in healthy individuals, and also in prostate cancer patients undergoing androgen deprivation [35], serum levels of IL-6, GH and IGF-1 increase [21], [22]. In the current study there is no detectable increase in serum IGF-1. This may be due to a transient mode of action that is already back to normal at two hours after exercise, when the current samples are taken.

Solid tumors consist not only of malignant epithelial cells. Growth and progression of tumors are dependent on a number of associated cell types such as fibroblasts, vascular and immune cells. The current study did not identify effects of exercise serum effect on the growth of fibroblasts. However, more studies are needed to evaluate the effect of exercise on the supportive role of the cells of the tumor microenvironment.

The current data show a robust effect of acute exercise on growth of an established prostate cancer cell line. In the future, follow up studies on the molecular basis for the differential effects of rest and exercise serum on prostate cancer cell growth are highly motivated, preliminary data suggest that compensating for the lower levels of EGF in the post exercise serum by adding rhEGF partly reverse the growth inhibitory effect of exercise serum (data not shown), thereby supporting the idea that multiple factors altered by exercise are involved. Furthermore, analyses of the effect of exercise serum on transformation of pre-malignant prostate epithelial cells would provide important additional information on the role of exercise in prostate cancer risk and progression.

In conclusion, despite the fear of possible detrimental effects of acute exercise serum on tumor cell growth, we show that even short-term effects seem to add to the overall beneficial influence of exercise on neoplasia.

## Supporting Information

Figure S1 Growth inhibition of prostate cancer cells by exercise serum is not mediated through induction of apoptosis. A) Flow cytometry of LNCaP cells stained with AnnexinIV and PI, squares giving distinct subsets of viable (low left), necrotic (top left), early (low right) and late (top right) apoptotic cells. B) Quantification of total (early apoptotic+late apoptotic) number of apoptotic cells after 24 hours incubation with rest and exercise serum, based on the number of hits in the different subsets defined in A). C) Quantification of total (early apoptotic+late apoptotic) number of apoptotic cells after 48 hours incubation with restand exercise serum based on the number of hits in the different subsets defined in A).
(PDF)

Figure S2 Growth inhibition of prostate cancer cells by exercise serum is not mediated through increased serum levels of cortisol.♮ denotes a significant (p<0.05) increase in s-cortisol directly after exercise. This increase has returned to normal levels in the exercise serum samples used in the analysis of prostate cancer cell growth (serum obtained 2 hours post exercise).
(PDF)
