# Supplementary material for: Effect of Acute Exercise on Prostate Cancer Cell Growth
Source: PLoS One. 2013 Jul 5;8(7):e67579. doi: 10.1371/journal.pone.0067579 (PMC3702495; doi:10.1371/journal.pone.0067579)
Supplement: Figure S1 — Growth inhibition of prostate cancer cells by exercise serum is not mediated through induction of apoptosis. A) Flow cytometry of LNCaP cells stained with AnnexinIV and PI, squares giving distinct subsets of viable (low left), necrotic (top left), early (low right) and late (top right) apoptotic cells. B) Quantification of total (early apoptotic+late apoptotic) number of apoptotic cells after 24 hours incubation with rest and exercise serum, based on the number of hits in the different subsets defined in A). C) Quantification of total (early apoptotic+late apoptotic) number of apoptotic cells after 48 hours incubation with restand exercise serum based on the number of hits in the different subsets defined in A). (PDF) [file pone.0067579.s001.pdf]

Supplementary figure 1

1A

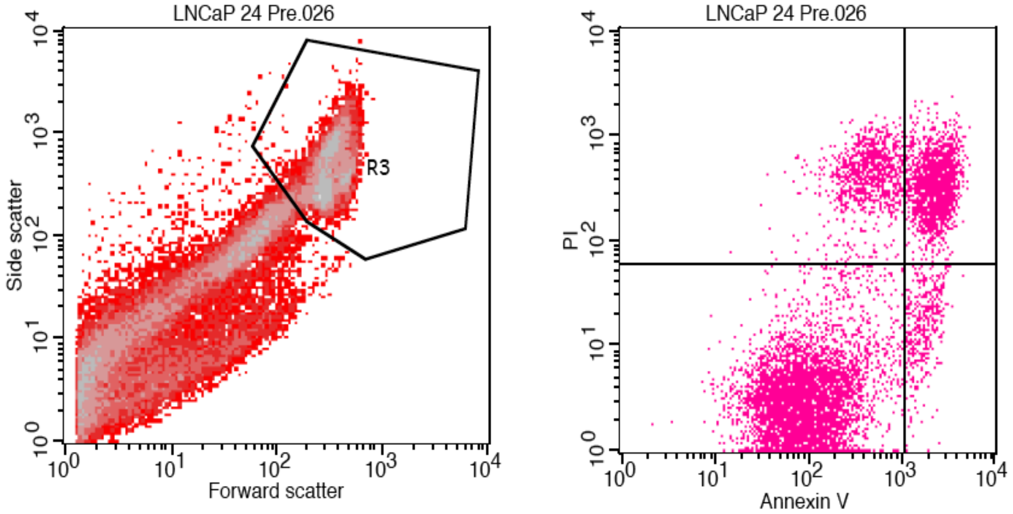

1B

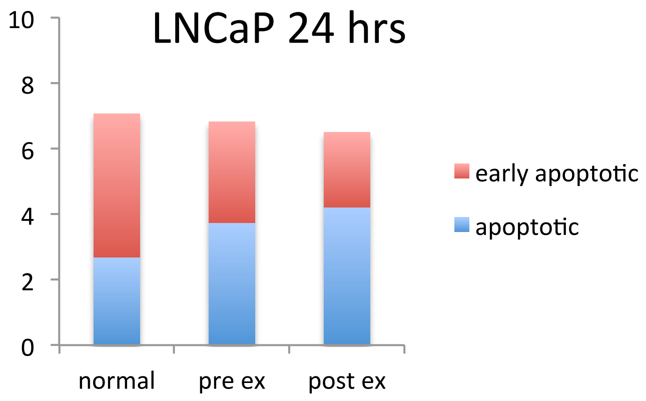

1C

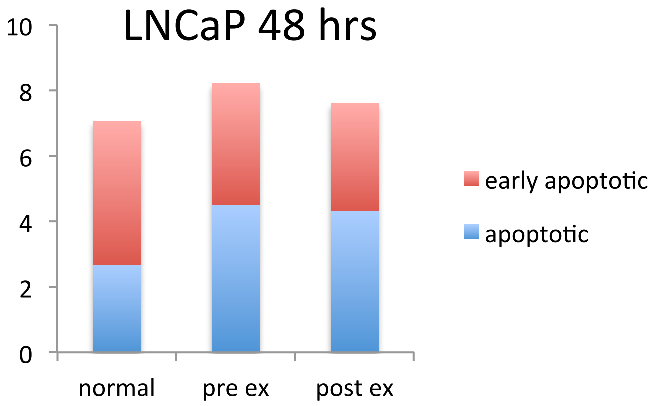

24 hrs

		normal	rest	exercise
necrotic	UL	6.27	5.67	5.9
apoptotic	UR	2.68	4.5	4.31
early apoptotic	LR	4.38	3.71	3.3
live	LL	86.66	86.12	86.5

48 hrs

		normal	rest	exercise
necrotic	UL	6.27	8.41	7.4
apoptotic	UR	2.68	3.73	4.21
early apoptotic	LR	4.38	3.09	2.28
live	LL	86.66	84.77	86.11
