# Supplementary figures and images for: Effect of Acute Exercise on Prostate Cancer Cell Growth
Source: PLoS One. 2013 Jul 5;8(7):e67579. doi: 10.1371/journal.pone.0067579 (PMC3702495; doi:10.1371/journal.pone.0067579)

## Supplementary figure 2

2A

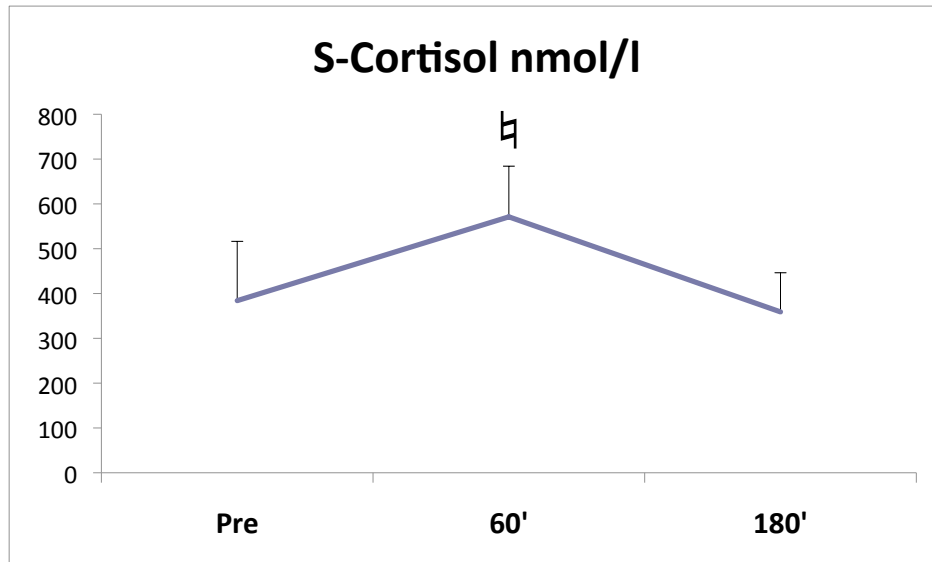

Supplement: Figure S2 — Growth inhibition of prostate cancer cells by exercise serum is not mediated through increased serum levels of cortisol.♮ denotes a significant (p<0.05) increase in s-cortisol directly after exercise. This increase has returned to normal levels in the exercise serum samples used in the analysis of prostate cancer cell growth (serum obtained 2 hours post exercise). (PDF) [file pone.0067579.s002.pdf]
